# Transmission of reduced levels of miR-34/449 from sperm to preimplantation embryos is a key step in the transgenerational epigenetic inheritance of the effects of paternal chronic social instability stress

**DOI:** 10.1080/15592294.2024.2346694

**Published:** 2024-05-13

**Authors:** Alexandre Champroux, Yang Tang, David A. Dickson, Alice Meng, Anne Harrington, Lucy Liaw, Matteo Marzi, Francesco Nicassio, Thorsten M. Schlaeger, Larry A. Feig

**Affiliations:** a Development, Molecular & Chemical Biology/Medical, Tufts University, Boston, MA, USA; b Stem Cell Program, Boston Children’s Hospital, Boston, MA, USA; c Tufts Graduate School of Biomedical Sciences, Tufts University School of Medicine, Boston, MA, USA; d Center for Molecular Medicine, MaineHealth Institute for Research, Scarborough, ME, USA; e Center for Genomic Studies, Instituto Italiano di Tecnologia Institution, Milan, Italy

**Keywords:** Epigenetic inheritance, miRNAs, sperm, chronic stress

## Abstract

The transgenerational effects of exposing male mice to chronic social instability (CSI) stress are associated with decreased sperm levels of multiple members of the miR-34/449 family that persist after their mating through preimplantation embryo (PIE) development. Here we demonstrate the importance of these miRNA changes by showing that restoring miR-34c levels in PIEs derived from CSI stressed males prevents elevated anxiety and defective sociability normally found specifically in their adult female offspring. It also restores, at least partially, levels of sperm miR-34/449 normally reduced in their male offspring who transmit these sex-specific traits to their offspring. Strikingly, these experiments also revealed that inducing miR-34c levels in PIEs enhances the expression of its own gene and that of miR-449 in these cells. The same induction of embryo miR-34/449 gene expression likely occurs after sperm-derived miR-34c is introduced into oocytes upon fertilization. Thus, suppression of this miRNA amplification system when sperm miR-34c levels are reduced in CSI stressed mice can explain how a comparable fold-suppression of miR-34/449 levels can be found in PIEs derived from them, despite sperm containing ~50-fold lower levels of these miRNAs than those already present in PIEs. We previously found that men exposed to early life trauma also display reduced sperm levels of miR-34/449. And here we show that miR-34c can also increase the expression of its own gene, and that of miR-449 in human embryonic stem cells, suggesting that human PIEs derived from men with low sperm miR-34/449 levels may also contain this potentially harmful defect.

The concept that the environment’s effects on an organism can be transmitted to offspring through epigenetic changes in germ cells is well established in organisms like C. *elegans* [1], drosophila [2] and plants [3]. More recently, evidence has emerged for this process occurring in male rodents, where many environmental exposures, including diet, drugs, temperature, enriched environment, and chronic stress, have been shown to affect the next generation(s) in the absence of any male input on rearing offspring [4]. Evidence for it also exists in humans, but it is mostly based on epidemiology [5–8]. The mechanisms underlying many of these phenomena in mammals are emerging, such as a role for environmentally induced changes in the content of specific sperm miRNAs in the transmission of stress-related traits across generations [9]. For example, studies on the effects of maternal separation [10], chronic unpredictable stress [11] and chronic mild stress [12] have shown phenotypes observed in offspring of these males can be mimicked by injection of pooled miRNA sample from stressed but not control mice into normal zygotes, which necessarily detects only miRNAs whose activities rise.

We have shown that exposing adolescent mice to chronic social instability (CSI) stress leads to reduced time spent in the open arms of the elevated plus maze and reduced interaction time with a juvenile mouse, phenotypes consistent with elevated anxiety and defective sociability respectively, specifically in female offspring for at least three generations through the paternal lineage [13]. Importantly, others subsequently confirmed our finding of female-specific transmission of these stress traits to offspring using a different mouse strain [11]. Then, we implicated CSI stress-induced reduction in sperm levels of members of the miR-34/449 miRNA family (miR-34b,c and miR-449a,b) in this process because their levels were all reduced not only in sperm of male mice exposed to this stress but also in sperm of their F1 male offspring who, while never being exposed to stress, transmit the anxiety and sociability defects specifically to their female offspring [14]. miR-34/449 miRNA family members are attractive candidates to transmit environmentally induced traits to offspring because they represent two of only 14 sperm miRNAs (out of hundreds) that are not expressed in oocytes [15,16].

Members of the miR-34/449 family of sperm miRNAs are also of particular interest because we subsequently found that multiple members of this family are also reduced in men who were raised in abusive and/or dysfunctional families as assessed by high scores on the Adverse Childhood Experience (ACE) survey [14]. And again, others subsequently confirmed the major aspect of this finding by demonstrating reduced levels of members of the miR-34/449 family in men who experienced early life trauma using a different assessment tool [17].

We also showed previously that the decrease in the content of members of both miRNA families in sperm persists after mating in preimplantation embryos, such that their levels are reduced ~ 5-fold through at least the morula stage [14]. This likely has significant effects on morula gene expression because miR-34/449 family members can inhibit expression of a common set of target mRNAs, predicted to include p53, CDK6, c-MYC, HDAC1, and BCL-2 [18–20], key regulators of gene expression.

Thus, we hypothesize that when their introduction into oocytes by sperm is suppressed when CSI stressed males’ mate, subsequent development is altered in a way that generates the stress-related phenotypes we detect in female offspring as well as reduced sperm miR-34/449 levels found in male offspring. To generate direct proof for the involvement of this family of miRNAs in this example of transgenerational epigenetic inheritance we developed a system to test their necessity described below.

## Materials and methods

### Animals and care facilities

All mice included in this study are of the CD-1 strain, obtained from Charles River Laboratories. Males used for CSI stress began the protocol at 28 days postnatal age, and control females used for breeding were 8 weeks of age. All animals are housed in temperature, humidity, and light- controlled (14 h on/10 h off LD cycle) rooms in a fully staffed dedicated animal core facility led by on-call veterinarians at all hours. Food and water were provided *ad libitum*. All procedures and protocols involving these mice were conducted in accordance with and approved by the Institutional Animal Care and Use Committee of the Tufts University School of Medicine, Boston, MA. No randomization procedures were needed to separate mice into groups.

### Tet-inducible miR-34c mouse

Using mouse genomic DNA, we synthesized custom primers to clone out the miR-34c gene, including ~ 100 bp of flanking gDNA on either side of the ~ 90 nt locus (which excludes miR-34b sequences) incorporating EcoRI and BamHI restriction enzyme sites to the 3’ and 5’ ends, respectively (Supp Fig. S1). This paradigm has been previously employed to express miR-34c for use in bovine embryos [21]. The miR-34c locus was ligated into the multiple cloning site (MCS) of the pTetOne plasmid (Takara Bio).

To pre-test the construct, the 34c-pTetOne plasmid was transfected into HEK293T cells and cultured with and without doxycycline (1 µg/mL) for 48 hours. Untreated cells transfected with 0.5 µg of plasmid showed only a 1.5-fold increase in miR-34c expression over untransfected cells, and doxycycline induced a close to ~ 200- fold increase in expression over baseline. Then the plasmid was purified, and linearized using the SspI restriction enzyme site. We injected the linearized plasmid into CD-1 mouse embryos to create our transgenic line. Ten founding animals were created from these injections, one of which displayed aggressive behaviour incompatible with mating, however 6 of the remaining 9 successfully transmitted this locus to the next generation of offspring, demonstrating germline integration of the transgene.

#### Chronic social instability (CSI) stress.

Starting at P28, the composition of each mouse cage (four mice per cage) was randomly shuffled twice per week, for 7 weeks, such that each mouse was housed with three new mice in a fresh, clean cage at each change as described [6,19]. Control mice were housed four mice per cage with the same cage mates for the duration of the protocol. After 7 weeks, mice were housed in pairs with a cage mate from the final cage change and left for 2 weeks to remove acute effects of the final change. Mice were then either sacrificed for sperm collection or mated with control female mice overnight to generate ‘F1’ animals, with successful mating confirmed via the presence of copulation plug the following morning. We have previously shown that stressed males can be mated multiple times and still transmit stress phenotypes to their offspring [13], so male mice were used for mating, embryo collection, or sperm collection as needed.

### Doxycycline treatment

After the CSI stress protocol was completed, males were mated with unstressed control WT females to generate ‘F1’ offspring or embryos at various stages. To avoid activating the gene in all cells of the male transgenic mice, we fed females doxycycline (40 mg/kg, Bio-serv, NJ, USA) 2-days before mating with the male and then doxycycline was removed from the chow.

### Mouse sperm collection

Mature, motile mouse sperm (from the cauda epididymis) was isolated via the swim-up method [22]. Briefly, male F0 and F1 mice were anesthetized under isoflurane and sacrificed via cervical dislocation. The caudal epididymis and vas deferens were dissected bilaterally and placed in 1 mL of warm (37 °C) M16 medium (Sigma-Aldrich, M7292) in a small Petri dish. Under a dissection microscope, sperm were manually expressed from the vas deferens using fine forceps, and the epididymis was cut several times before incubating at 37°C for 15 min to allow mature sperm to swim out, then large pieces of tissue were removed. The remainder of the extraction took place in a 37°C warm room. The sperm-containing media was centrifuged at 3,000 RPM for 8 min, supernatant was withdrawn and discarded, and 400 μL of fresh, warm M16 medium was then carefully placed on top of the pellet. The tubes were then allowed to rest at a 45° angle for 45 min to allow the motile sperm to swim-up out of the pellet into the fresh medium. The supernatant containing the mature sperm was then carefully withdrawn and centrifuged again for 5 min at 3,000 RPM to pellet the motile sperm. Supernatant was withdrawn and discarded, and the pellet was frozen on dry ice for later processing.

### Mouse embryo collection

To obtain sufficient numbers of embryos, a superovulation protocol was employed. Control female mice were injected with 5 U of pregnant mare serum gonadotropin (PMSG, nor272-Prospecbio, Israel) and 46 h later injected with 5 U of human chorionic gonadotropin (HCG, C1063 Sigma-Aldrich) and placed into the home cage of the male to mate overnight. In the morning, female mice were checked for copulation plugs and returned to their own cages. If morula-stage embryos were desired, female mice were sacrificed about 2 days later. On the day of collection, mice were sacrificed via cervical dislocation. Under a dissection microscope, the uterus was removed, and a very high-gauge needle was inserted into the opening of the fallopian tubes and the embryos were flushed out using warm 37°C M2 medium (Sigma-Aldrich, M7167). The embryos were separated according to cell number, and any unfertilized ova or embryos displaying unusual morphology were discarded. The embryos of interest were pooled (~40–60) by cell stage and individual, and snap frozen on dry ice. As the females are sacrificed during the collection process, this entire protocol was repeated with new females for every embryo stage collection to generate one pool each of one specific stage embryo for both F0 control fathers and F0 stressed fathers CSI WT and Tg and with or without doxycycline.

### CHB-4 human embryonic stem cells and miRNA mimic transfection

hESC line CHB-04 [23], on the NIH registry of approved hESC lines, was transitioned from MEF-dependent culture to feeder-free culture using mTeSR® medium (Stem Cell Technologies) and 6-well plates coated with hESC-qualified Matrigel® (Corning) for 1 h at 37°C. One passage prior to transfection, the cells were single-cell passaged using TrypLE Select Enzyme (Gibco) and replated onto Matrigel in mTeSR® supplemented with 10 µM Y-26732 (Tocris) for 24 hrs. to improve cell survival and attachment. Cells were grown with 5% CO2 at 37°C. Cells were tested for mycoplasma regularly.

For transfection, CHB-04 cells were cultured to 70% confluency in a 6-well plate. Each miRNA (mimic miR-34c, 50pmole, #MC11039, or mimic negative control, #4464058, Thermofisher) was added to 5 l R buffer (Invitrogen) and incubated for 10 minutes prior to transfection. CHB-04 cells were transfected using the Neon electroporator (Invitrogen) using the Neon 10 µL transfection kit. About 400,000 single-cell dissociated CHB-04 cells were resuspended in 35 μl R buffer and added to the miRNA suspensions. The CHB-04 cells were then loaded into a 10 µL Neon tip and electroporated using the following pulse conditions: 1200 V 30 ms 1 pulse. Cells were replated into one well of a Matrigel®-coated 6-well plate in mTeSR® medium as before and cultured for 2 days after the initial transfection.

### RNA lymphocytes collection and doxycycline treatment

Under isoflurane anaesthesia, a small incision at the end of the tail of mice was made and blood was collected into a tube containing EDTA. Then, 1 ml of lysis buffer was added and incubated at room temperature for 5 min. The solution was transfered to a 15 ml canonical tube and 13 ml of PBS containing 0.4% EDTA and 0.1% BSA was added and centrifuged at room temperature at 350×g for 5 min. The supernatant was removed and 200 µl complete media was added at 4°C. Half of each was treated with doxycycline 1.5 µg or PBS for 24 h at 37°C. RNA was then extracted using the Norgen RNA Isolation kit (#51800, Norgen Biotek Corp.) according to the manufacturer’s protocol and the concentration of RNA was determined using a NanoDrop-1000 before qPCR miR-34/449 familly analysis (Thermofisher Scientific).

### RNA tissue collection

Total RNA from male liver and testis was extracted using the miRNEasy Micro kit (#1071073), Qiagen.

### RNA extraction and quantitative real-time PCR

Total RNA was extracted from both mouse sperm samples and human embryonic stem cells, using the miRVana Isolation Kit (Invitrogen, #AM1561) according to the manufacturer’s protocol (Total RNA isolation). Total RNA was extracted from mouse embryos using the Norgen RNA Isolation kit (#51800, Norgen Biotek Corp.) according to the manufacturer’s protocol. The concentration of RNA was determined using a NanoDrop-1000 (Thermofisher Scientific). Relative gene expression for all miRNAs samples was determined using the Taqman Advanced cDNA synthesis and qPCR system (Applied Biosystems).

An aliquot of 10 ng of total RNA from sperm, or embryos was used in the initial cDNA synthesis step. Real-time PCR was performed for each target in duplicate on a StepOnePlus PCR System (Applied Biosystems) with primers from TaqMan™ Advanced miRNA Assay (#A25576, Thermofisher, miR-34c-5p, #478052_miR, miR-449a, #478561_miR, miR-34b, #478049_miR, miR-449b, #479528_miR, miR-34a, #478048_miR, miR-152, #477921, miR-375 #mmu481141_miR). Relative gene expression for all Pri- forms was determined using the High-Capacity cDNA Reverse transcription kit and qPCR system (Applied Biosystems) Pri-miRNA primers for mouse or human (TaqMan™ Pri-miRNA Assay, #4427012, mouse Pri-34bc Mm03306660_pri, mouse Pri-449ab Mm03308638_pri, human Pri-34bc Hs03295169_pri, human Pri-449ab Hs03304853_pri) were used.

All data was analysed using the Comparative ΔΔCT method [24,25] to generate relative expression data using miR-192-5p (#A25576, Thermofisher, #478262_miR) as the internal control for miRNAs since its Ct values did not change significantly enough after of any of our experimental manipulations to affect conclusions (see Supplemental Fig. S5 and supplemental Tables 1–6) and GAPDH Forward 5’-CATGTTCCAGTATGACTCCACTC-3,’ Reverse 5’-GGCCTCACCCCATTTGATGT-3;’ for mouse Pri- and ACTB (Forward 5-‘AGAAAATCTGGCACCACAC-3,“ Reverse 5”-TAGCACAGCCTGGATAGCAA −3’) for human ES cells samples [24].

**Table 1.** Bestkeeper, NormFinder and geNorm analysis on three different candidate miRNA internal controls.

miRNAs	192	152	375
geo Mean [Ct]	27.3	27.93	23.63
AR mean [Ct]	27.31	27.96	23.8
min Cq [Ct]	25.96	25.67	20.24
max Cq [Ct]	29.12	31.51	30.71
std dev [± Ct]	0.59	1.08	2.29
CV [% Ct]	2.17	3.88	9.63
stability value	0.871	1.451	2.954
M stability value	1.692	1.692	2.688

**Table 2.** Bestkeeper, NormFinder and geNorm analysis on three different candidate miRNA internal controls.Geo Mean = geometric Mean of Ct values are Mean = Arithmetic mean of Ct values, stdev = standard deviation of Ct values, CV= coefficient variation.

	Control Tg		CSI Tg no Dox		CSI Tg Dox	
miRNAs	Mean Ct	Stdev	Mean Ct	Stdev	Mean Ct	Stdev
192	26.919	1.033	27.391	0.203	27.869	0.628
152	27.506	1.153	28.155	2.001	28.498	1.277
375	24.947	3.269	22.091	2.014	23.681	2.004

**Table 3.** Bestkeeper, NormFinder and geNorm analysis on three different candidate miRNA internal controls. Geo Mean = geometric Mean of Ct values are Mean = Arithmetic mean of Ct values, stdev = standard deviation of Ct values, CV= coefficient variation.

	Control Tg		CSI Tg no Dox		CSI Tg Dox	
miRNAs	Mean Ct	Stdev	Mean Ct	Stdev	Mean Ct	Stdev
192	26.919	1.033	27.391	0.203	27.869	0.628
152	27.506	1.153	28.155	2.001	28.498	1.277
375	24.947	3.269	22.091	2.014	23.681	2.004

**Table 4.** Bestkeeper, NormFinder and geNorm analysis on three different candidate miRNA internal controls. Geo Mean = geometric Mean of Ct values are Mean = Arithmetic mean of Ct values, stdev = standard deviation of Ct values, CV= coefficient variation.

	Control WT		CSI WT no Dox		CSI WT Dox	
miRNAs	Mean Ct	Stdev	Mean Ct	Stdev	Mean Ct	Stdev
192	25.777	0.825	25.020	1.634	26.019	1.388
152	26.197	1.716	25.967	2.174	26.579	2.037

**Table 5.** Bestkeeper, NormFinder and geNorm analysis on three different candidate miRNA internal controls. Geo Mean = geometric Mean of Ct values are Mean = Arithmetic mean of Ct values, stdev = standard deviation of Ct values, CV= coefficient variation.

	Control WT		CSI WT no Dox		CSI WT Dox	
miRNAs	Mean Ct	Stdev	Mean Ct	Stdev	Mean Ct	Stdev
192	25.777	0.825	25.020	1.634	26.019	1.388
152	26.197	1.716	25.967	2.174	26.579	2.037

**Table 6.** Bestkeeper, NormFinder and geNorm analysis on three different candidate miRNA internal controls. Geo Mean = geometric Mean of Ct values are Mean = Arithmetic mean of Ct values, stdev = standard deviation of Ct values, CV= coefficient variation.

	Control WT		CSI WT no Dox		CSI WT Dox	
miRNAs	Mean Ct	Stdev	Mean Ct	Stdev	Mean Ct	Stdev
192	25.777	0.825	25.020	1.634	26.019	1.388
152	26.197	1.716	25.967	2.174	26.579	2.037

### Mouse behavior

Adult F1 female mice derived from control and stressed males mated to both females fed normal or doxycycline-containing chow were tested in the elevated plus maze (EPM) and then direct social interaction with a juvenile tests done 1 week apart. All behavioural tests were carried out during the light phase of the cycle, and mice were acclimated to the testing room 1 h prior to any behavioural procedure.

#### Elevated plus maze (EPM) test

Mice were placed in the centre of a plus-shaped maze elevated 40 cm from the floor, composed of two open and two closed arms, each 35.5 cm long and 5 cm wide (Campden Instruments Ltd, Lafayette, IN). General mouse activity was analysed for 5 min, and percent of time spent in the open arms was recorded using the Ethovision XT (Noldus, Leesburg VA, USA).

#### Direct social interaction with a juvenile (SIJ) test

Direct social interaction was assessed in a clean cage like the home cage in which the animals were regularly housed, with bedding on the floor, and covered by a plastic lid to avoid direct light exposure. Each experimental mouse was placed into this cage and left to habituate for 15 min. Then, an unfamiliar same-sex juvenile (26–28 days old) was introduced and left there for 3 min. Testing sessions were recorded and videos were analysed by an observer (blind to the treatments) taking only the experimental animal into account. During the 3 min test, time spent performing the following affiliative and social behaviours was measured: sniffing the juvenile, grooming it, following it, crawling over or under it, doing passive physical contact with it, and walking side by side.

### Statistical analysis

GraphPad Prism 9.0 software package was used for analysing the behavioural and qPCR data. For all experiments comparing two groups, unpaired t-test was used. For all experiments comparing more than two groups, ordinary one-way ANOVA with multiple comparisons with Tukey test were used. *p* < 0.05 was used as a cut-off for significance. Outliers were detected and removed according to the ROUT outlier test (with Q = 1%) [26].

## Results

### Transgenic mouse system demonstrates the necessity for reduced miR-34/449 levels found in preimplantation embryos derived from CSI stressed males in propagating stress-related phenotypes to offspring

Others studying different chronic stress paradigms have implicated different sperm miRNA changes in generating stress-related phenotypes in offspring by injecting pools of miRNAs they observed rise in sperm of stressed males into control zygotes. They observed phenotypes in offspring generated from these zygotes that were similar to those observed by mating stressed males, confirming their importance to those processes [10,11]. However, altered phenotypes were not observed when individual miRNAs were injected. This implied that multiple miRNAs were necessary to induce a phenotype, which prevented the analysis of how specific miRNAs contribute to the process.

**Figure 1.**
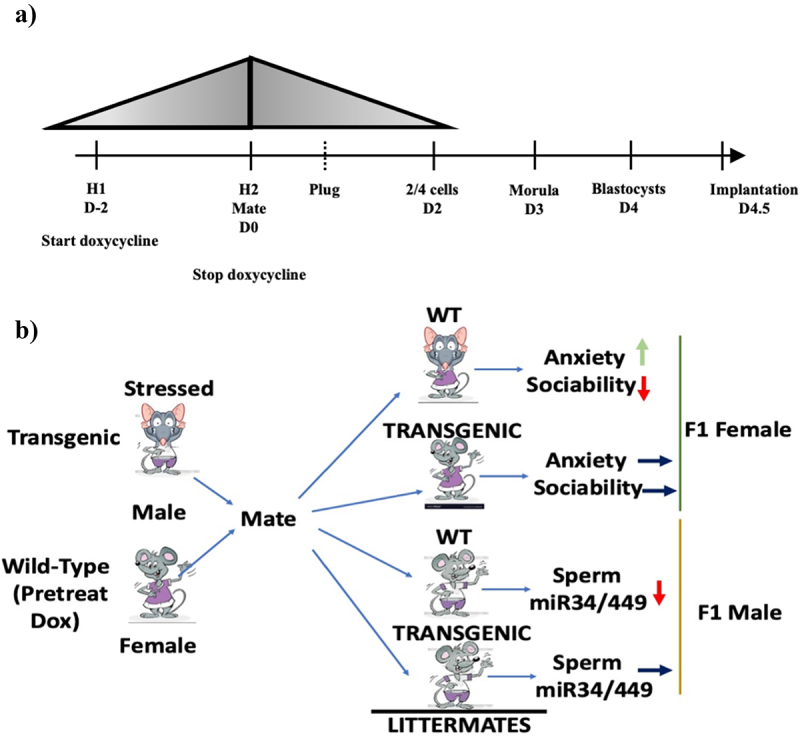
Transgenic mouse model for detecting involvement of altered preimplantation miRNA content in transmitting traits across generations. a) timeline describing transient doxycycline (dox) introduction into WT female mice. Dox (40 mg/kg) was added to female mouse chow 48 hrs. before mating and then removed at mating. Its concentration in animals estimated (grey triangle based on known 48 hr t ½ of dox). b) scheme for how mating CSI-stressed, heterozygous transgenic, males with females pretreated with doxycycline can test whether reduced preimplantation embryo miR-34/449 levels participate in generating stress-related behaviours in female and suppressed sperm miR-34/449 levels in male offspring. WT: wild-type.

Because we were interested in revealing the specific role of the miR-34/449 family in the transgenerational epigenetic inheritance of the effects of CSI stress, we tested its necessity rather than sufficiency by designing a method to test if restoring reduced levels of miR-34/449 present in **p**re**i**mplantation **e**mbryos (**PIEs**) derived from mating CSI stressed males prevents transmission of stress-related traits across generations. To this end, we generated tet-inducible miR-34c transgenic mice and mated transgenic males with wild-type females fed doxycycline for 2 days before mating (see overall scheme in Figure 1a and details in Methods and **Supplemental. Fig. S1**). In this way, miR-34c was transiently induced only in PIEs beginning two days after fertilization when endogenous embryo genes become active. Because the transgenic males are heterozygous, half the embryos were experimental transgenics and half wild-type controls (Figure 1b). Another advantage of this system over directly injecting miRNAs into zygotes is that future experiments revealing how these miRNAs contribute to development involve setting up new matings rather than new rounds of collecting zygotes, microinjections, and implantations into foster mothers.

**Figure 2.**
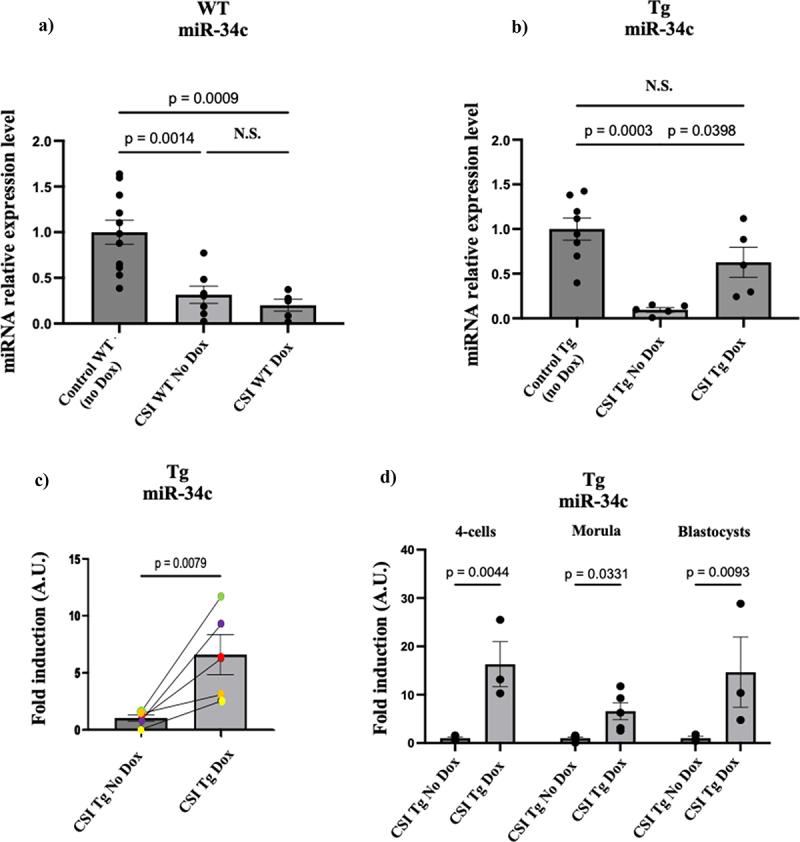
Induction of miR-34c levels in preimplantation embryos derived from mating CSI-stressed, tet-inducible miR-34c transgenic males. a) relative miR-34c levels in morula stage embryos derived from mating either unstressed WT males to females fed normal chow (first bar) or CSI-stressed WT males each mated to both females fed regular chow (second bar) or Dox (third bar). *n* = 11 for control WT, *n* = 6 for CSI WT No Dox and *n* = 5 for CSI WT Dox. Dox: doxycycline. Data are expressed as mean ± S.E.M. One-way Anova, multiple comparisons. N.S.= not significant. b) miR-34c level in morula stage embryos derived from mating either unstressed transgenic males to females fed normal chow (first bar) or CSI-stressed transgenic (Tg) males each mated to both females fed normal chow (second bar) or Dox (third bar). *N* = 8 for control WT, *n* = 5 for CSI WT No Dox and *n* = 5 for CSI WT Dox. Dox: doxycycline. Data are expressed as mean ± S.E.M. One-way Anova, multiple comparisons, N.S.= not significant. c) 5 independently derived CSI-stressed transgenic (tg) males were mated to both WT unstressed females pre-fed doxycycline (Dox) or regular chow (no Dox) and the fold-change in miR-34c level were measured in morula stage embryos derived from each mating. Data are expressed as mean ± S.E.M; Mann-Whitney test two-tailed, N.S.= not significant. c) miR-34c induction from the 2–4 cells stage through the blastocyst stage of preimplantation embryos derived from CSI stressed, tet-inducible transgenic males mated to both females pre-fed a control or dox-containing chow. Pools of 40–60 embryos were isolated at the indicated times. *n* = 3 for 4 cells and blastocysts and *n* = 5 for morula. Data are expressed as mean ± S.E.M; Mann-Whitney test two-tailed, N.S.= not significant. In all cases, miR-192 is used as an internal assay control because we found its ct values did not change significantly after any of the perturbations tested (see Supplemental Fig. S5).

Figure 2 shows that the system worked as expected. Doxycycline pretreatment of females had no effect on miR-34c levels in morula derived from CSI stressed wild-type males (Figure 2a) but did induce miR-34c levels on average ~ 4-fold in morula derived from mating 5 independent transgenic males each to both normally fed and Dox-fed females (Figure 2b). Since the morula collected from mating transgenic fathers was a mixture of WT and transgenic embryos the induction shown is an underestimate of ~ 2-fold. Figure 2c shows miR-34c induction in morula by individual transgenic males to both control and Dox-treated females used to generate Figure 2b. Figure 2d shows induction (with no dox treatment of each set at 1) occurred as early as the 4-cell stage and persisted through the blastocyst stage of embryogenesis.

**Figure 3.**
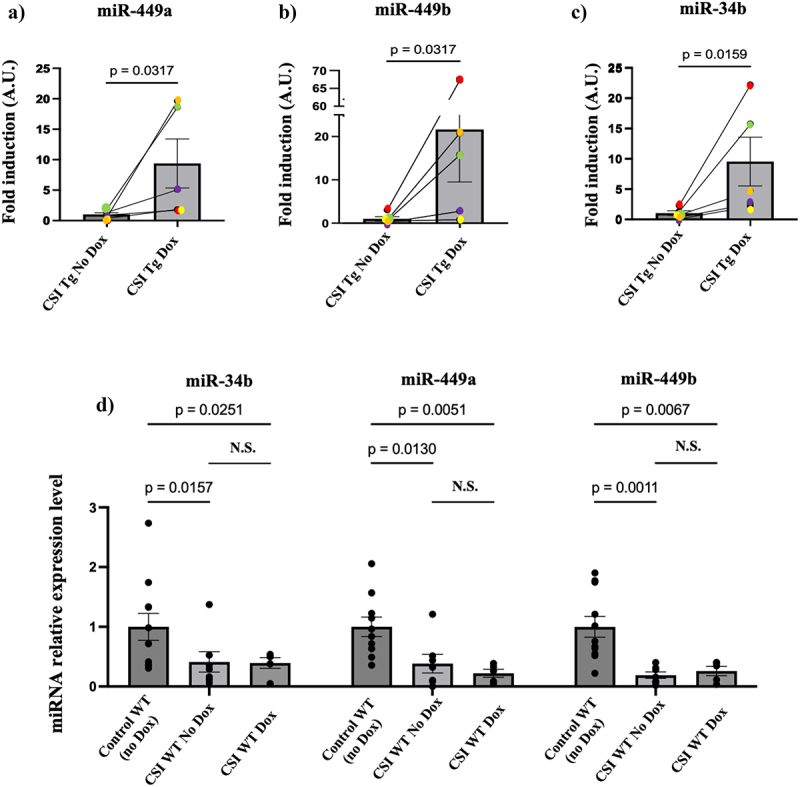
Induction of miR-34c levels in morula stage mouse embryos derived from mating CSI stressed transgenic but not WT males, increases the levels of miR-449a/b and miR-34b in them. a-c) 5 independently derived CSI-stressed transgenic (Tg) males were mated with both WT unstressed females pre-fed regular chow (no Dox) or doxycycline (Dox) and the fold change in levels of miR-34/449 family members levels were measured in morula stage embryos derived from these mating. Data are expressed as mean ± S.E.M; Mann-Whitney test two-tailed, d) miR-34/449 family levels in morula stage embryos derived from mating CSI stressed WT males with WT unstressed females fed with regular chow or doxycycline. Data are expressed as mean ± S.E.M; two-way ANOVA multiple comparison, N.S.= not significant.

When we designed these mice, we assumed that because miR-34c levels are much higher than miR-449 levels in PIEs that its restoration would be sufficient to rescue at least some of the phenotypes found in offspring. But when we compared their levels in morula from these rescued PIEs we noticed that in each of the transgenic males miR-449a and miR-449b levels (measured with a probe with no sequence similarity to miR-34c) were also elevated by pretreating mothers with doxycycline even though both isoforms of miR-449 are expressed from a different gene (Figure 3a,b). To test whether exogenous miR-34c induces expression of its own gene in morula we could not assay miR-34c directly since we can’t distinguish the endogenous gene product from the transgenic one. So instead, we measured miR-34b, which is a splice variant generated from the same pri-miRNA using a probe from a region of miR-34b with no homology to miR-34c. And just as we found for miR-449a/b, induction of miR-34c increased the levels of miR-34b in morula (Figure 3c). Dox did not have an independent effect on these miRNAs, because if the transgene was not present in morula, as when WT CSI stressed males were used instead of transgenic males, pre-feeding females with it had no effect on any of these miRNAs (Figure 3d). Moreover, the effect of miR-34c induction on other miRNAs showed specificity of the system since the levels of miR-375 and miR-152, two miRNAs we found unchanged in sperm of male mice exposed to CSI stress [14] were not altered (Supplemental Fig. S2).

Elevating expression of a gene does not usually enhance the level of a redundant family member, as it could threaten homoeostasis. In fact, the opposite normally occurs for miR-34/449. For example, previous studies have shown that in a miR-449 gene knockout mouse expression of miR-34a,b rises [18]. Moreover, we noticed that when we induced miR-34c expression in lymphocytes isolated from transgenic males by exposing them to doxycycline *in vitro*, it suppressed miR-34b levels (Supplemental Fig. S3). Thus, this amplification of the miR-34/449 family by miR-34c phenomenon may be unique to early embryos, and likely explains how the very low levels of miRNAs like miR-34/449 found in sperm can have large effects on their levels in early embryos to induce a phenotype (see Discussion).

### Reduced levels of miR-34/449 detected in PIEs derived from mating CSI stressed males are necessary for the transmission of indicators of elevated anxiety and defective sociability to their female offspring

**Figure 4.**
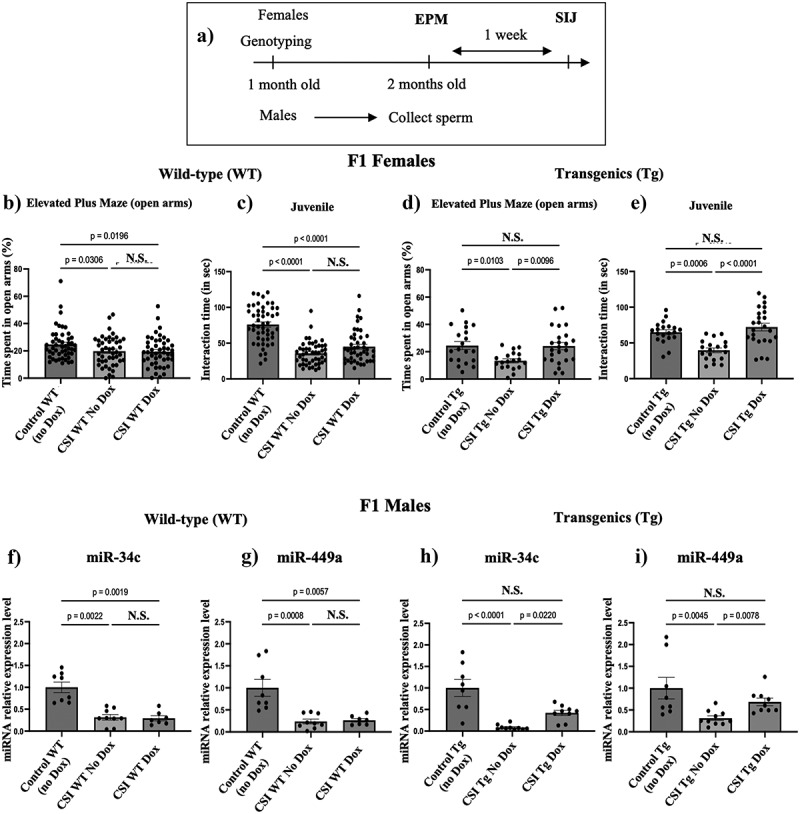
Restoring miR-34c/449 levels in PIEs derived from mating CSI-stressed males suppresses stress -associated phenotypes, and at least partially prevents reduced miR-34c and miR-449a levels in sperm normally found when these embryos mature into female and male adults, respectively. a) timeline for assessing stress-related phenotypes in F1 wild-type and transgenic female and male littermates of control and CSI stressed males. b and d) measurement of the % time mice spent in the open arms of the EPM for WT and transgenics (Tg) littermates derived from control or CSI-stressed transgenic males mated to either control-fed or dox pre-fed females. c and e) interaction time in social interaction with juvenile assay for WT and transgenics (Tg). Control WT *n* = 50, CSI WT No dox *n* = 39, CSI WT dox *n* = 44, control Tg *n* = 20, CSI Tg No Dox *n* = 19 and CSI Tg Dox *n* = 24. Data are expressed as mean ± S.E.M. Ordinary one-way Anova with multiple comparisons (Tukey correction). N.S.= not significant. d, e) measurement of miR-34c and miR-449a in sperm of male WT and transgenic (f, g) littermates of females analysed in B and C above. Control WT *n* = 8, CSI WT No Dox *n* = 9, CSI WT Dox *n* = 7, control Tg *n* = 8, CSI Tg No Dox *n* = 10 and CSI Tg Dox *n* = 9. Expression was normalized to that for miR-192. Data are expressed as mean ± S.E.M; Kruskal-Wallis test with multiple comparisons, dox: doxycycline, tg: transgenic, WT: wild-type. N.S.= not significant.

Since we could rescue both miR-34 and miR-449 levels in morula from CSI stressed transgenic males, in a new set of experiments we used the same induction protocol described in Figure 1a but let offspring of the set of unstressed and CSI-stressed transgenic males develop into adults, and separately assayed offspring by sex and genotype (WT vs transgenic) (see Figure 1b). We then compared WT and transgenic adult female littermates of unstressed and stressed transgenic fathers for: i) the % time they spent in the open arms of the EPM (as an indicator of anxiety), and ii) their interaction time with a juvenile (SIJ as an indicator of sociability) (Figure 4a).

As expected from our previous findings [13], WT female offspring of CSI stressed transgenic fathers mated to control-fed females spent less time in open arms (Figure 4b), and interacted less with a juvenile (Figure 4c) than WT females from unstressed transgenic fathers, and pre-feeding mothers with doxycycline (Dox) had no effect in either case (Figure 4b,c). Also as expected, transgenic female offspring of CSI stressed transgenic fathers mated to control-fed mothers also spent less time in the open arms of the Elevated Plus Maze (Figure 4d) and interacted less with a juvenile than those from unstressed transgenic fathers (Figure 4e). However, both stress-related phenotypes returned to normal levels in transgenic female offspring derived from mating the same CSI stressed transgenic fathers to Dox pre-fed mothers (Figure 4d,e) (see females in right side of Figure 1b).

### Reduced levels of miR-34/449 detected in PIEs derived from mating CSI stressed males are also necessary for the transmission of reduced sperm miR-34/449 to their male offspring

To test whether restoring levels of miR-34/449 in these PIEs prevents the appearance of reduced levels of these miRNAs found in their male offspring, we compared the sperm miR-34c (Figure 4f,h) and 449a (Figure 4g,i) levels in the same sets of offspring as described for females above, but this time in males (see males Figure 1b right). As expected from our previous results, sperm of WT male littermates derived from mating CSI transgenic stressed males to control-fed mothers contained reduced levels of both miR-34c and miR-449a, compared to sperm of WT male offspring derived from mating these mothers with non-stressed transgenic fathers, and this was not altered when these CSI stressed transgenic males were mated with Dox pre-fed females (Figure 4f,g). In contrast, while transgenic male littermates derived from CSI stressed transgenic fathers mated to control fed mothers also showed reduced sperm levels of both miR-34c and miR-449a, levels of both miR-34c and miR-449a returned, at least in part, to control levels when the same fathers were mated to Dox pre-fed mothers (Figure 4h,i) (see males in right side of Figure 1b).

### miR-34c regulates the expression of its own gene products and those for miR-449 in both mouse and human early embryo cells at a posttranscriptional level

**Figure 5.**
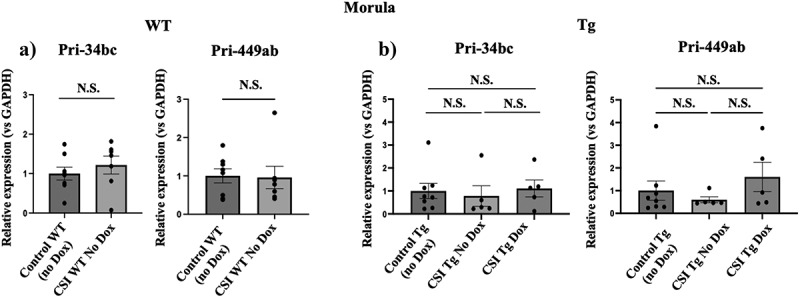
miR-34c alters the level of miR-449a,b in morula stage mouse embryos at the post-transcriptional level. a) levels of Pri-34bc and Pri-449ab were measured in morula stage embryos derived from the mating of unstressed and stressed WT, Mann-Whitney test, control WT *n* = 8, CSI WT No dox *n* = 6 for Pri-34bc and control WT *n* = 8, CSI WT No Dox *n* = 6 for Pri-449ab, WT: wild-type, CSI: chronic social instability. b) levels of Pri-34bc and Pri-449ab were measured in morula stage embryos derived from the mating of unstressed, stressed and stressed inducible miR-34c transgenic males. In the experiments, the CSI F0 fathers are the same between no dox and dox groups. Expression was normalized to that for GAPDH. Data are expressed as mean ± S.E.M, Kruskal-Wallis test with multiple comparisons. Dox: doxycycline, Tg: transgenic, CSI: chronic social instability. Control Tg *n* = 8, CSI Tg No Dox *n* = 5 and CSI Tg Dox *n* = 5. N.S.= not significant.

To begin to understand how miR-34c influences expression of its own gene and that for miR-449 in early embryo cells, we compared the levels of the primary miRNA transcripts (Pri-miRNA) of miR-34 and miR-449 that generate miR-34b,c and miR-449a,b respectively as an indicator of transcription rate, in morula from control and CSI stressed WT fathers (see processing steps for miR-34b,c and miR-449a,b,c in Supplemental Fig. S1C). The levels of neither of these Pri-miRNAs changed (Figure 5a) despite their mature levels declining ~ 5-fold (see Figure 3d).

Moreover, the Pri-miRNA levels of neither miR-34b,c nor miR-449a,b rose in morula derived from stressed transgenic fathers mated to dox pre-fed mothers (Figure 5b), despite the mature levels of both miRNAs rising ~ 5-fold (see Figures supplemental S2B and Figures 3A-C respectively). This implies that miR-34c normally positively regulates members of its own gene and that of miR-449 in PIEs at a step downstream of transcription, likely by suppressing its degradation, and this effect is suppressed when males are exposed to CSI stress. Levels of miR-34c and miR-449a are not always regulated at the post-transcriptional level since in testes, where expression of both is known to be high and liver where they are known to be low, the levels of the primary form of these miRNAs match their fully processed levels (see Supplemental Fig. S4).

**Figure 6.**
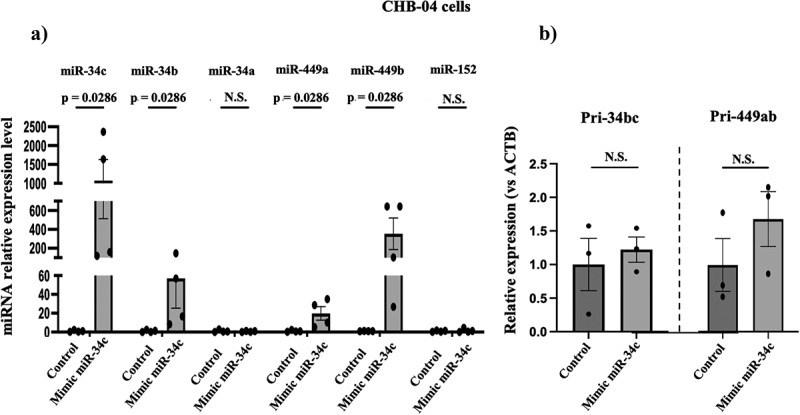
Elevation of miR-34c levels in human embryonic stem cells, increases the levels of miR-34b and miR-449a and b in them, but not at the transcriptional level. a) CHB-04 human embryonic stem cells were transfected with a random control miRNA (control) or miR-34c mimic 48 hrs. later the levels of miRnas indicated were assayed. *n* = 4. Data are expressed as mean ± S.E.M; Mann-Whitney test two-tailed, b) Pri-34bc and Pri-449ab levels were measure 48 hrs after transfecting CHB-04 human embryonic stem cells, expression was normalized to that for ACTB. Data are expressed as mean ± S.E.M. Mann-Whitney test two tailed, *n* = 3 N.S.= not significant.

We [14] and then others [17] showed that men’s exposure to early life trauma leads to reduced sperm miR-34c levels when they are adults. If this leads to reduced PIE levels of miR-34/449 in humans as it does in mice, it will raise the possibility that men with reduced sperm miR-34c levels might put offspring at risk for stress-related disorders. To begin to test this idea, we transfected CHB-04 human embryonic stem cells with miR-34c and a scrambled control mimic, and found the former led to a robust enhancement of expression of miR-34b (but not miR-34a which is expressed from a different genetic locus nor miR-152, neither of which is altered in sperm by CSI stress) and miR-449a and b (Figure 6a). Moreover, despite dramatically increasing levels of miR-34b and miR-449a human embryonic stem cells, no effect was observed on the levels of their Pri-forms (Figure 6b). Thus both in mouse and human early embryo cells miR-34c regulates members of its own gene’s expression and that of its close relative miR-449 at a posttranscriptional level that likely involves miRNA stability via target-directed degradation [27], a topic presently under investigation.

## Discussion

Two major conclusions can be derived from this work. First, we had previously demonstrated strong correlative evidence that reduced levels of miR-34/449 in sperm and preimplantation embryos derived from them participate in generating phenotypes observed in male and female offspring of CSI stressed males. Now, we provide direct evidence by showing that restoring to normal suppressed levels of miR-34/449 in preimplantation embryos derived from mating CSI stressed males prevents the appearance of elevated anxiety (as measured with the Elevated Plus Maze) and defective sociability (by measuring interaction time with a juvenile) normally found when they mature in adult females. It also restores, at least in part, reduced miR-34/449 levels normally found when male preimplantation embryos mature into adults.

Second, these studies suggest a mechanism by which environmentally induced changes in the very small amounts of miRNAs present in sperm can, after fertilization, promote significant changes in the content of much higher levels of miRNAs found in in preimplantation embryos. In particular, we found that the levels of miR-34/449 in sperm are about 50-fold lower than those normally present in 2–4 cell embryos, which makes it difficult to understand how reductions in sperm levels added to oocytes upon fertilization can make a functional difference on embryos unless some type of amplification of the sperm effect occurs in preimplantation embryos.

Amplification mechanisms solving this dilemma have been identified and shown to be necessary for epigenetic inheritance in organisms like C. *elegans* [1] and plants [3], but until now no analogous systems have been revealed in mammals [28,29]. In fact, the lack of such evidence has promoted scepticism that transgenerational epigenetic inheritance exists in mammals [5,30]. The mechanism we describe is based on the observation that the miR-34/449 levels normally present in preimplantation embryos are dependent upon the delivery from sperm of miR-34c, because when sperm levels of miR-34b,c and miR-449a,b are suppressed in CSI-stressed males, expression of both genes are suppressed in preimplantation embryos. But when miR-34c levels are restored in these PIEs expression from both miR-34b and miR-449a,b genes are restored as well in these early embryo cells. Interestingly, we find the regulation of both by miR-34c is post-transcriptional, either at the miRNA processing step or at the level of miRNA stability, an issue presently under investigation.

How do these results relate to studies on other forms of chronic stress that induce different phenotypes in offspring that are mediated by changes in different sperm miRNAs? These include chronic variable stress that induces a suppressed HPA axis response in offspring [11], early maternal separation that yields depressive-like phenotypes [10] and chronic mild stress [12] that promotes depressive like phenotypes in offspring. Each of these paradigms has common features that are distinct from those of CSI stress, which suggests they may require distinct amplification mechanisms. First, although many environments besides CSI stress also generate reduced levels of specific sperm non-coding RNAs [12,31–34], the stress studies mentioned above all implicate sperm miRNAs whose levels rise in response to stress [10,11,12]. Second, those studies have not shown that miRNA changes detected in sperm persist in preimplantation embryos derived from them. Finally, while one of the three paradigms, maternal separation [10], generates multigenerational behaviour effects, it does not show consistent sperm changes in F1 males as we do for CSI stress.

The difference in the ability to detect miRNA changes in early embryos derived from stressed males may be related to whether the sperm miRNA levels fall or rise, rather than the specific stress paradigm. That is because we reported previously that in addition to sperm miR-34/449 levels falling in sperm of CSI stressed males, miR-409-3p levels rise across generations [35], yet we do not detect a commensurate rise in its level in early embryos (data not shown). Others have speculated that the increased levels of miRNAs they detect in sperm from stressed mice can nonetheless impact zygotes significantly by excessively suppressing maternal mRNAs. This is because while the maternal versions of these miRNAs are present in oocytes, they are inactive until the 2-cell stage when their endogenous counterparts begin being expressed [36]. However, changes in maternal mRNAs in zygotes from stressed mice have yet to be documented to play a role in the process. Another possibility is that these miRNAs are covalently modified in sperm to magnify their activities above those of their endogenous early embryo counterparts upon fertilization, a phenomenon proposed for tRNA fragments involved in mediating the effects of diet across generations [37–39].

Reduced insertion of sperm miR-34/449 into oocytes after mating CSI stressed males could also potentially impact maternal RNAs in zygotes before endogenous versions of these miRNAs are expressed. However, this cannot be sufficient for the offspring phenotypes we observe. This is because in our miR-34c transgenic mouse system restoration of miR-34/449 levels in preimplantation embryos from CSI stressed transgenic mice only begins at the 2-cell stage when endogenous genes, including the miR-34c transgene, become activated. Yet it prevents the appearance of the altered phenotypes investigated here in both female and male offspring. Thus, maintained suppression of the miR-34/449 family while endogenous miRNAs are actively expressed in preimplantation embryos beginning at the 2-cell stage is necessary for the phenotypes induced by CSI stress in both male and female offspring to fully appear.

Of potential translational significance is our finding of a similar post-transcriptional miRNA amplification mechanism in human embryonic stem cells. This is because we showed previously that the levels of miR-34/449 are also reduced in sperm from men exposed to severe adverse childhood experiences [14]. Thus, findings presented here raise the possibility that preimplantation embryos derived from these men also display reduced levels of these miRNAs. Since we showed here that this perturbation contributes to the transmission of stress-related phenotypes from CSI treated male mice to their female offspring, it may also contribute to the negative effects these types of experiences are known to have on their offspring [40,41], in this case females. And because the perturbation also contributes to reduced sperm miR-34/449 levels in F1 male mice, it may also cause epigenetic inheritance to be transgenerational in humans.

Further investigation of this possibility is of particular interest because susceptibility to mental health disorders contains a significant inherited component [42]. And a recent mathematical model based on existing clinical evidence supports the idea that a fraction derives from epigenetic inheritance [43], which theoretically is easier to reverse than genetic inheritance.

## Supplementary Material

Supplemental Material

## Data Availability

The data that support the findings of this study are available from the corresponding author upon reasonable request.
