# Supplementary material for: Transmission of reduced levels of miR-34/449 from sperm to preimplantation embryos is a key step in the transgenerational epigenetic inheritance of the effects of paternal chronic social instability stress
Source: Epigenetics. 2024 May 13;19(1):2346694. doi: 10.1080/15592294.2024.2346694 (PMC11093028; doi:10.1080/15592294.2024.2346694)
Supplement: Supplemental Material [file KEPI_A_2346694_SM8309.zip › Supplementary files/Supplemental_Figure_& tables legends.docx]

**Supplemental Figure Legends**

**Supplemental Figure 1: Map Of the pTetOne plasmid containing the miR-34c locus and flanking genomic DNA.** Mmu-miR-34c genomic locus and ~100bp of flanking genomic DNA on either side of the locus were cloned into the pTetOne vector using the BamHI and EcoRI restriction enzyme sites. TREGs-insert-FWD and Tet-insert-REV primers used for sequencing to original confirm successful integration of the miR-34c locus into the plasmid and are also used for genotyping the transgenic animals. B) Genomic region of miR-34b/c in the mouse genome on the chromosome 9. C) Expression of miR-34c and miR-34b. The miRNAs are produced together in a primary transcript, Pri-34b/c, from the region chromosome 9 (51,014,134:51,015,000:-1). Then the Pri-34b/c is cleaved into Pre-34b and Pre-34c before being exported to the cytoplasm, where final maturation into miR-34c and miR-34b takes place ^44^(A similar process occurs for miR-449a and b).

**Supplemental Figure 2:** Levels of miRNAs unrelated to CSI stress are not altered by CSI stress nor by miR-34c induction in morula stage embryos derived from CSI stressed transgenic males.

**A)** Levels of miR-375 and miR-152 in morula derived from mating unstressed and stress transgenic males. Data are expressed as mean ± S.E.M; One-way Anova, multiple comparison. N=6 for control and n=5 for CSI Tg.

B) 5 different CSI stressed transgenic (Tg) males were mated with WT unstressed females pre-fed doxycycline (Dox) or with regular chow (no Dox) and measure of miR-375 and miR-152 in morula stage embryos derived from these mating. n=5. Data are expressed as mean ± S.E.M; Mann-Whitney test two-tailed. n.s= not significant

**Supplemental Figure 3: miR-34b levels are suppressed in transgenic lymphocytes cells when miR-34c levels are elevated by their exposure to doxycycline.** Lymphocytes were isolated from Tet-inducible miR-34c transgenic blood, and then exposed to either doxycycline or saline for 24h. While miR-34c content was increased by doxycycline, miR-34b content decreased. miR-449a/b was undetectable in both cases. n=5. Data are expressed as mean ± S.E.M; Unpaired t test two-tailed, n.s= not significant

**Supplemental Figure 4:** Levels Pri- and mature forms of miR-34b,c and miR-449a,b are consistent with each other in testis and liver.

**A**) Levels of Pri-34b/c and miR-34b and miR-34c in liver vs testis.

**B)** Levels of Pri-449a/b and miR-449a,b in liver vs testis.

N=2, Internal standards for the Pri-miRNAs were GAPDH, and miR-192 for the mature form.

**Supplementary Figure 5- Quantification cycles of internal normalizers coupled to results of BestKeeper, NormFinder and geNormanalysis for miRNA internal controls^45^.**

**A**) Quantification cycles of miR-192 in transgenics morula (graph) and Bestkeeper, NormFinder and geNorm analysis on three different candidate miRNA internal controls (Tables 1 and 2).

**B)** Quantification cycles of miR-192 in wild-type morula (graph) and Bestkeeper, NormFinder and geNorm analysis on three different candidate miRNA internal controls (Tables 3 and 4).

**C) Quantification** cycles of miR-192 in ES cells (graph) and Bestkeeper, NormFinder and geNorm analysis on three different candidate miRNA internal controls (Tables 5 and 6).

**D)** Quantification cycles of miR-192 in transgenics sperm.

**E)** Quantification cycles of miR-192 in wild-type sperm.

**F)** Quantification cycles of miR-192 in lymphocytes.

Geo Mean = geometric Mean of Ct values are Mean = Arithmetic mean of Ct values, stdev = standard deviation of Ct values, CV= coefficient variation.  Data are shown as mean of Ct values +/- SEM. One-way Anova with multiple comparisons for 3 groups and T-test for the ES cells and lymphocytes.

**Supplementary Tables 1-6: Quantification cycles of internal normalizer coupled to results of BestKeeper, NormFinder and geNormanalysis for miRNA internal controls^45^.**

**Tables 1 and 2:** Bestkeeper, NormFinder and geNorm analysis on three different candidate miRNA internal controls.

**Tables 3 and 4:** Bestkeeper, NormFinder and geNorm analysis on three different candidate miRNA internal controls.

**Tables 5 and 6:** Bestkeeper, NormFinder and geNorm analysis on three different candidate miRNA internal controls.

Geo Mean = geometric Mean of Ct values  are Mean = Arithmetic mean of Ct values, stdev = standard deviation of Ct values, CV= coefficient variation.
