# Supplementary figures and images for: Transmission of reduced levels of miR-34/449 from sperm to preimplantation embryos is a key step in the transgenerational epigenetic inheritance of the effects of paternal chronic social instability stress
Source: Epigenetics. 2024 May 13;19(1):2346694. doi: 10.1080/15592294.2024.2346694 (PMC11093028; doi:10.1080/15592294.2024.2346694)

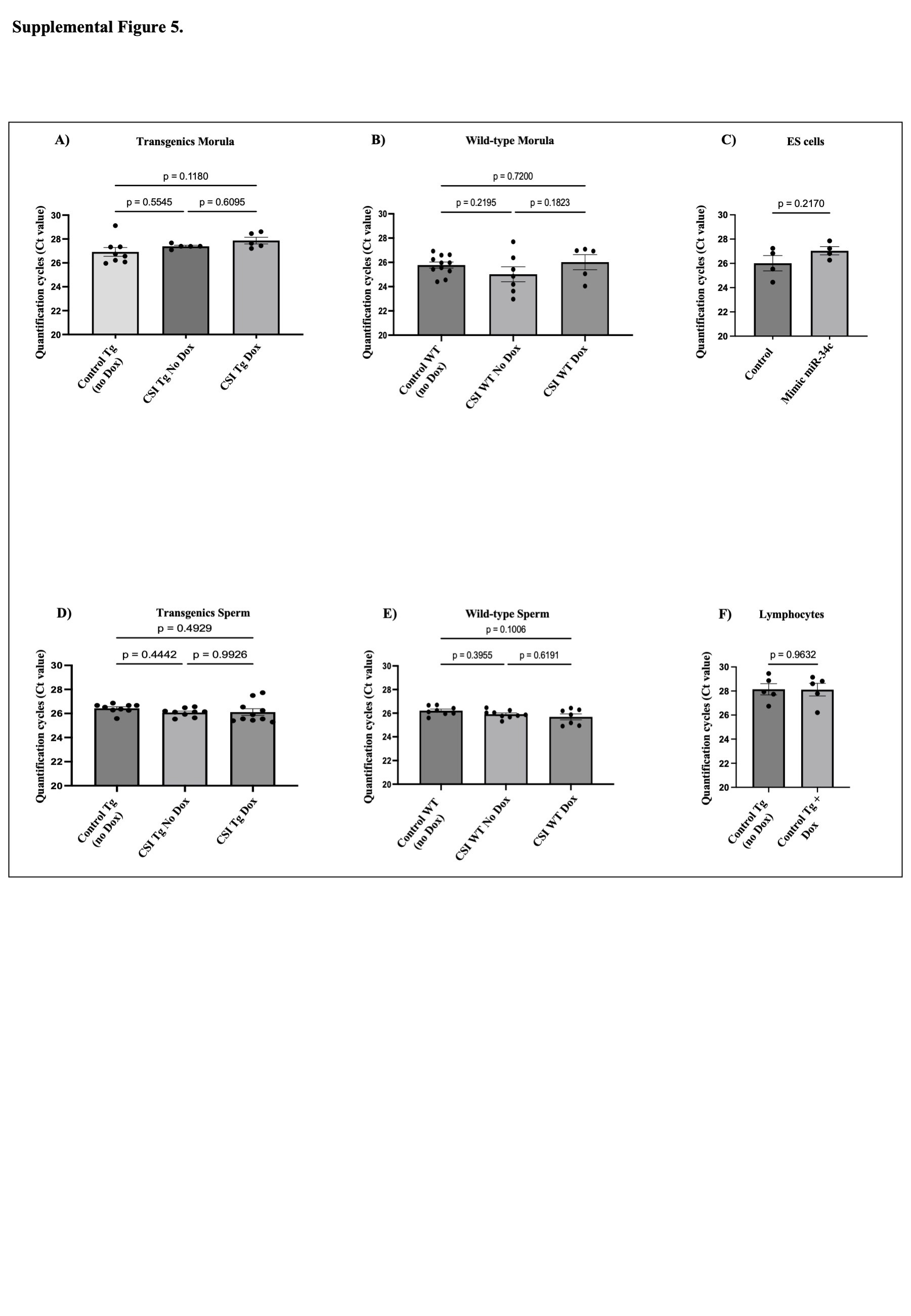

Supplement: Supplemental Material [file KEPI_A_2346694_SM8309.zip › Supplementary files/New_supp_fig_5.jpg]

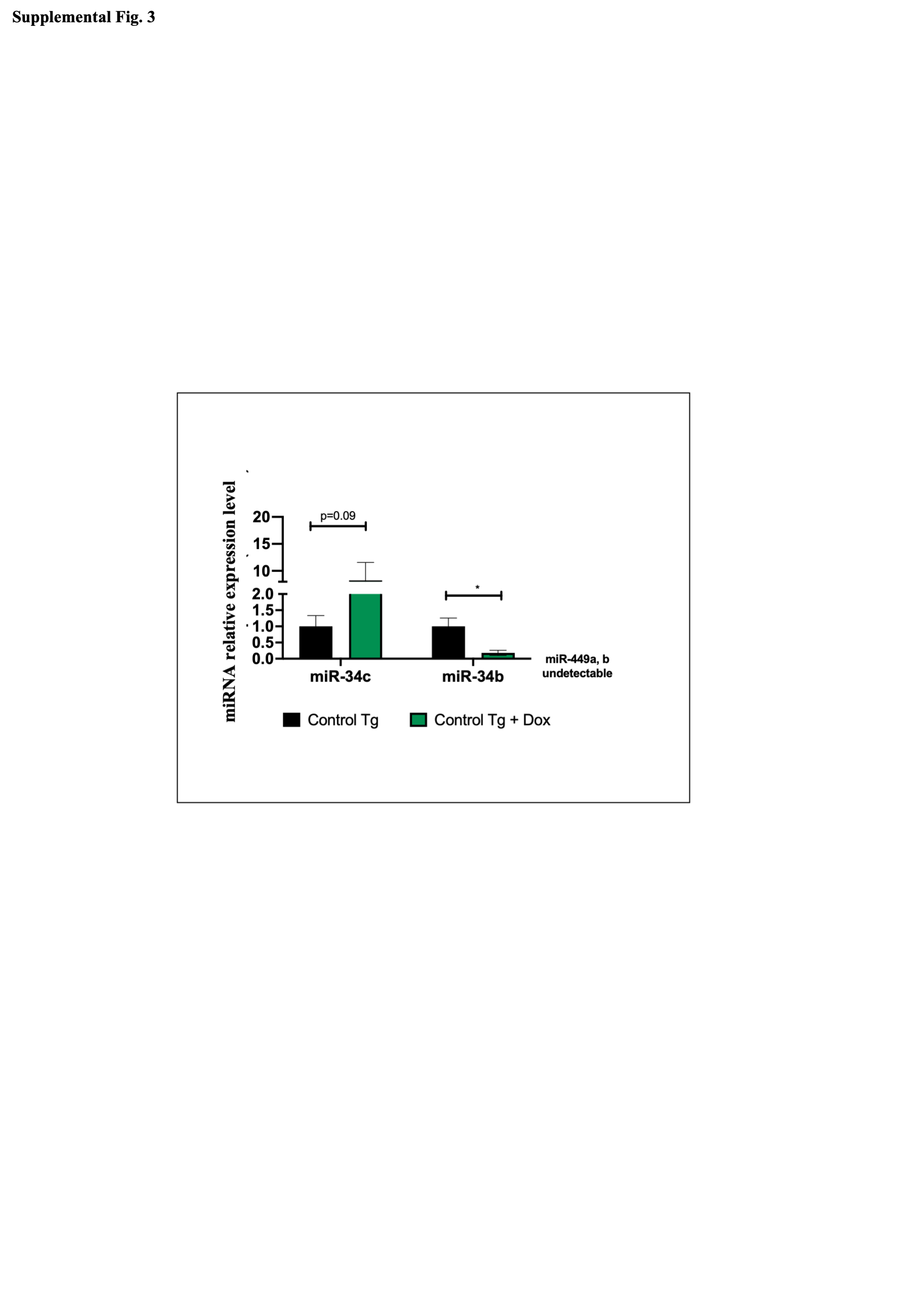

Supplement: Supplemental Material [file KEPI_A_2346694_SM8309.zip › Supplementary files/Slide10 (Supp Fig 3).tiff]

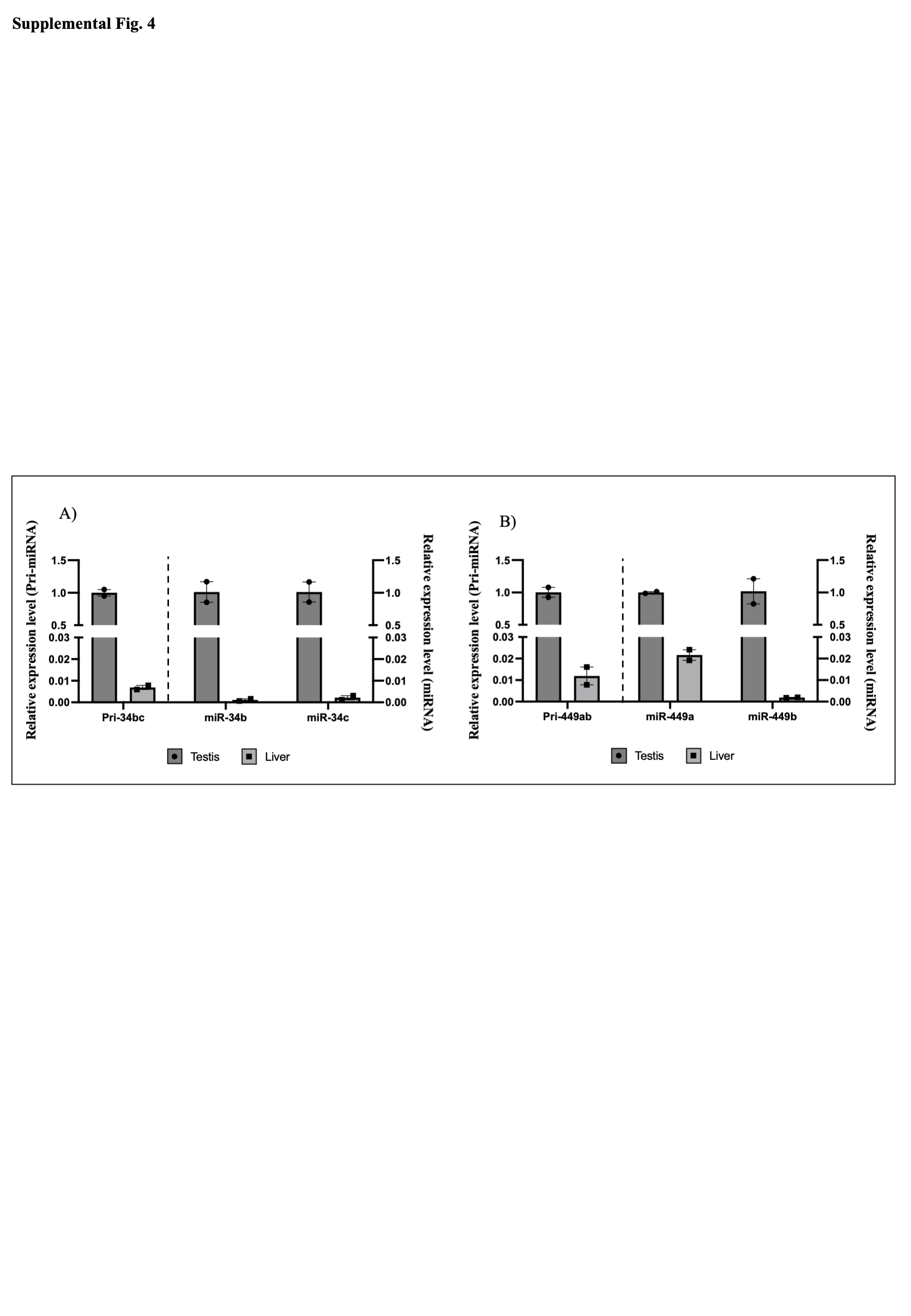

Supplement: Supplemental Material [file KEPI_A_2346694_SM8309.zip › Supplementary files/Slide11 (Supp fig 4).tiff]

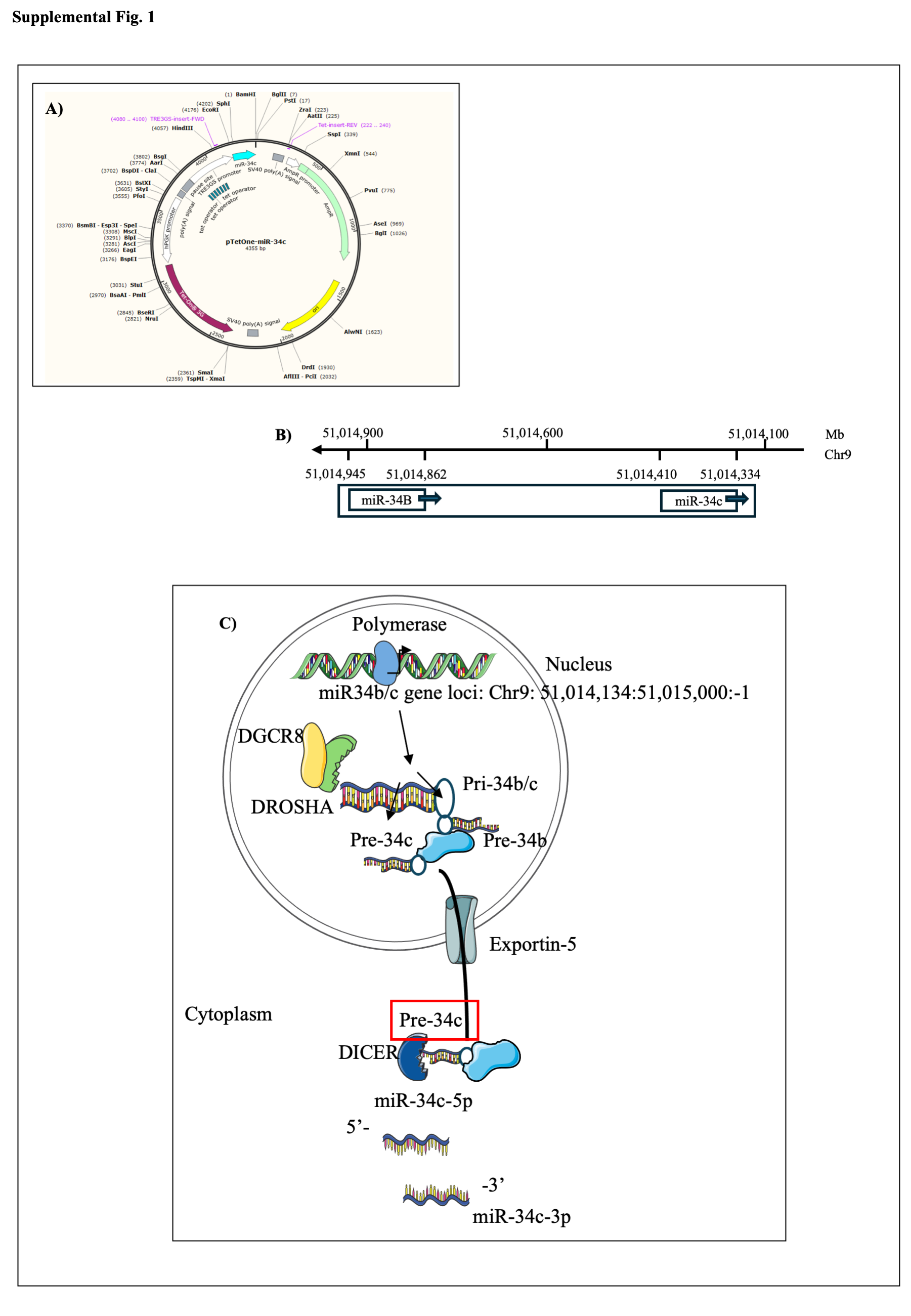

Supplement: Supplemental Material [file KEPI_A_2346694_SM8309.zip › Supplementary files/Slide8 (Supp fig 1).tiff]

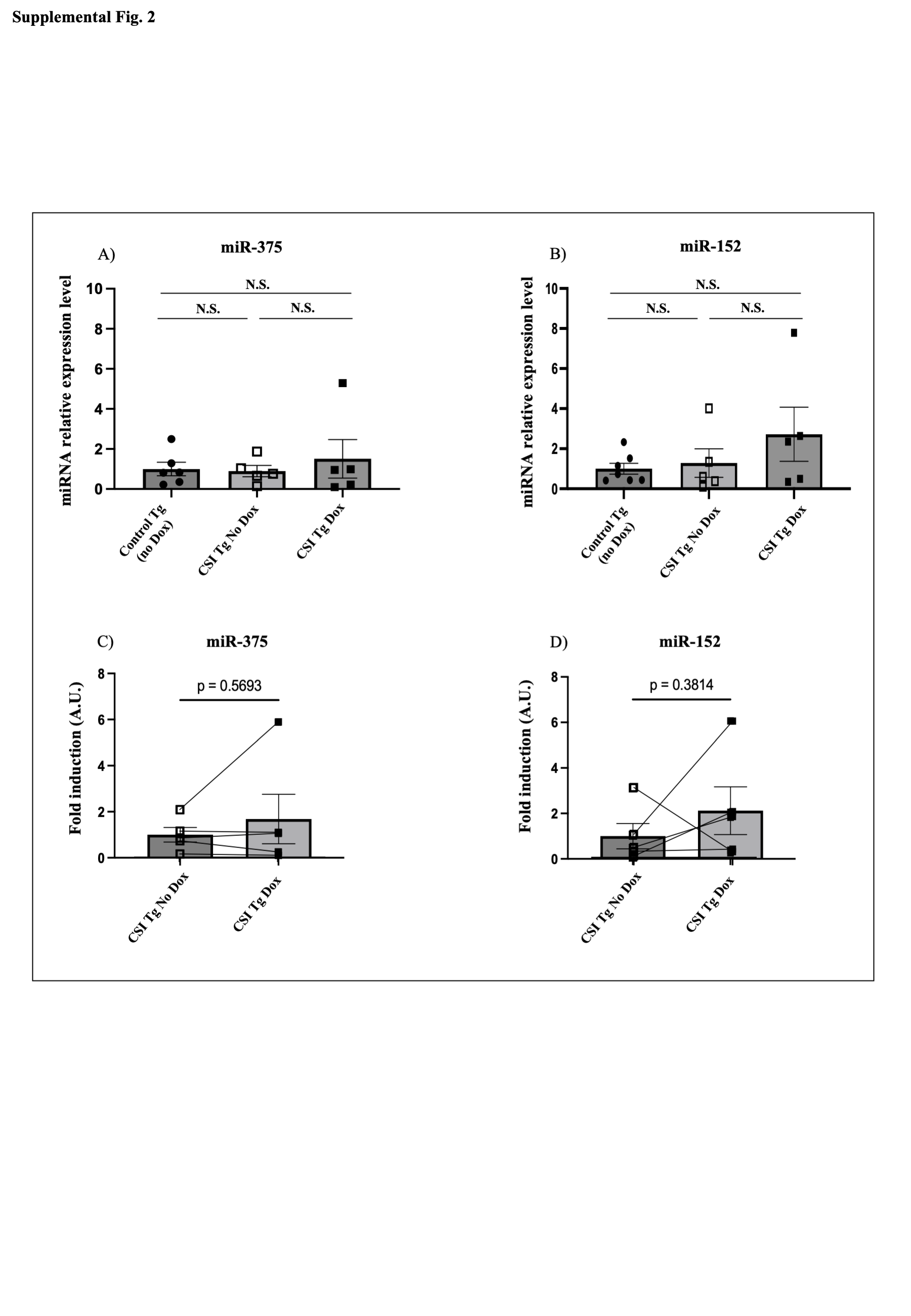

Supplement: Supplemental Material [file KEPI_A_2346694_SM8309.zip › Supplementary files/Slide9 (Supp Fig 2).tiff]
